# Pesticide usage and occupational hazards among farmers working in small-scale tomato farms in Cameroon

**DOI:** 10.1186/s42506-019-0021-x

**Published:** 2019-06-18

**Authors:** Ayuk B. Tambe, Baleba M. R. Mbanga, Dapi L. Nzefa, Medoua G. Nama

**Affiliations:** 1 Centre for Food and Nutrition Research, Institute of Medical Research and Medicinal Plant Studies (IMPM), MINRESI, P.O. Box 13033, Yaoundé, Cameroon; 2 Division of Human Nutrition, Department of Global Health, Stellenbosch University, P.O. Box 241, Cape Town, South Africa; 3 Department of Social Work, Faculty of Social Sciences, Linnaeus University, Växjö, Sweden

**Keywords:** Tomato farmers, Pesticides, Occupational health and safety, Cameroon

## Abstract

**Background:**

Agriculture is undoubtedly the backbone of the Cameroonian economy, and other economic activities thrive only if production in this sector is assured. It has been estimated that approximately 25 million agricultural workers worldwide experience unintentional pesticide poisoning yearly. Unfortunately, limited information exists about the health and safety of the farmers. The aim of this study was to describe the occupational health and safety (OHS) conditions of farmers working on small-scale tomato farms in the western region of Cameroon.

A cross-sectional research method was used to collect data from tomato farmers in May 2017, using a questionnaire developed by the research team.

**Results:**

A total of 104 tomato farmers from small-scale farms participated in the study. The analysis revealed that the occupation is male-dominated (86.5%). The training and use of personal protective equipment (PPE) among farmers were rare (35.6%), and farmers were mostly exposed to chemical hazards. The farmers reported the following work-related health problems: skin irritation, backache, impairment of the central nervous system (CNS), visual problems, and respiratory difficulties.

**Conclusions:**

The OHS conditions on small-scale tomato farms are mostly poor, thus predisposing farmers to the risk of work-related health problems. Exposure to occupational hazards can be significantly reduced if the required PPE are available and efficiently used.

## Introduction

Agriculture is a vital economic sector in Cameroon, especially since an estimated 45% of Cameroon’s gross domestic product (GDP) depends on it [1]. The traditional food crop is part of an integrated household-farming system [2]. Therefore, crop destruction would paralyse many households; subsequently, farmers rely on pesticide use for pest control due to its assumed lower cost. However, according to the World Health Organization (WHO) standards [3], only pesticides that are safe to farmers and farm-workers should be used.

Pesticide use has increased over the past 20 years, the highest identified in low-income countries starting from a low base such as Cameroon, Ethiopia, and Burkina Faso with an 8- to 50-fold increase [4]. In middle-income countries like China, Argentina, Brazil, and Thailand pesticide use increased from three- to eightfold while it has been stable, or even decreasing, in high-income countries like in the USA, Germany, Japan, and Denmark [4]. Even though there has been a rise in pesticide use in developing countries, very limited information exists about the health and safety of the farmers.

Pesticides used on tomato farms are classified according to their target organisms, chemical class, and toxicity. According to their target organism, they are divided into insecticides, fungicides, herbicides, rodenticides, and bactericides. Previous studies have revealed that the most used pesticides in low-income countries like Cameroon are insecticides, contrary to herbicides which are mostly used at the global level, given that weeding is done manually in Cameroon [5–8]. According to their chemical classes, the most common pesticides used are organophosphates, organochlorides, carbamates, pyrethroids, and dipyridils.

WHO has divided pesticides into the toxicity classes Ia, Ib, II, III, U in decreasing toxicity, and O being the sign for obsolete pesticides. Obsolete pesticides are defined as those pesticides that can no longer be used for their intended purpose and therefore must be disposed of. These include, among others, banned, outdated, and deteriorated pesticides according to the Food and Agriculture Organization (FAO) [9]. Although a great number of toxic pesticides under the WHO classes Ia and Ib and some pesticides belonging to class II and class O have been restricted for use in several countries, they are still extensively used in middle- and low-income countries. The use of these restricted pesticides is due to its broad spectrum application, costs, and ease of use [6]. The intensive use of pesticides on tomato farms seems to ensure the best-quality produce which guarantees good prices for farmers and excellent sales for vendors [10].

Work-related pesticide poisoning has increased globally, especially in less-developed countries [11]. It has been estimated that yearly about 25 million agricultural workers globally experience unintentional pesticide poisoning [12]. The main obstacle to control and prevent work-related pesticide poisoning is that the scope and magnitude of this issue often remains uncharacterised, especially in an underserved population such as farmers [13]. In Cameroon, pesticide-poisoning data are often reported as incidence data from hospitals. Contrary to workers in large agricultural companies who may receive safety training on the use of pesticides to reduce exposure, the majority of tomato farmers in Cameroon work independently, only having small plots of farmland. They may apply pesticides using simple backpack style applicators without adequate knowledge of the basic safety measures.

The aim of this study was to describe the occupational health and safety (OHS) conditions in the use of pesticides among farmers working in small-scale tomato farms in the western region of Cameroon. The specific objectives of the research were to collect the demographic profile of the farmers, identify the training and use of personal protective equipment (PPE), and report work-related complaints among small-scale tomato farmers.

## Materials and methods

### Survey participants

A quantitative cross-sectional survey was conducted to collect data from 104 small-scale tomato farmers relating to their demographic profile, OHS knowledge and practice on the use of pesticides, and the occupational health complaints. The farmers were interviewed at their various farms to conduct an on the spot inspection on the use of pesticides.

### Study area

The western region is a major tomato-growing region in Cameroon, and the overuse of pesticides to manage pests and diseases has been observed. Although these chemicals increase crop yields, they can also cause health and environmental hazards when used irresponsibly.

### Data collection

The data collection was done in May 2017 by the research team. The farmers were interviewed at farms, in French language. Inclusion criteria were tomato farm workers living in the western region of Cameroon and working in small-scale farms in 2017. A total of 106 tomato farmers were eligible for the study. A snowball sampling method was used to gather information from all eligible participants. In this case, referrals were asked from already identified participants, given the lack of database, as most of the workers are presumably unregistered.

Data were collected through a well-structured interviewer-led questionnaire and on-site observations to complement responses got from the use of the questionnaire. The questionnaire consisted of three sections, namely the demographic profile, farmers’ OHS knowledge and practice on the use of pesticides, and the occupational health complaints.

The demographic profile included age, gender, marital status, education level, and work experience. The knowledge and practice of participants were measured through seven items relating to the training on the use of pesticides, safety boots, safety glasses, gloves, a nose mask, raincoats, and cleaning the body immediately after the use of pesticides. In this scale, each item had more than one correct answer, and all the correct answers in each item were summarized to give one point. Thus, the total score of knowledge and practice ranged from 0 to 7. Regarding occupational health complaints, five questions were asked concerning the following items: skin irritation, backache, nervous system injury, visual problems, and respiratory difficulty. Occupational health complaints were evaluated based on a 2-point Likert scale (yes = 1 and no = 0). The occupational health complaints’ score ranged from 0 to 5.

The farmers were interviewed in their various farms to conduct an on the spot inspection on the use of pesticides. The questionnaire was pre-tested on five farmers to ensure the language used was appropriate and could be understood by all farmers. The feedback was incorporated into the final questionnaire used in this study.

### Data analysis

The data collected was coded, entered, cleaned, and analysed using Epi Info version 7. The frequencies were established for categorical data. Univariable analysis using the chi-square test to verify the relationship between the safe use of PPE and the gender of the farmers was used. The Pearson chi-square test was used mainly due to the fact that the dependent or independent variables had two categories. The accepted level of significance for determinants of practice of OHS was set at 0.05.

## Results

A total of 106 tomato farmers were initially surveyed and interviewed. Two questionnaires were rejected since the participants did not complete the questionnaires as required. The results reported are based on the data collected from 104 tomato farmers.

### Demographic details

The current study analysis indicated that the average age of the participants was 38.0 ± 10.3 years. The majority of the farmers were between 31 and 40 years, and of that group, 86.5% was male. Most of the participants were married (81.7%) and had at least secondary education (62.5%). The average work experience of the farmers was 4.8 ± 1.2 years with the majority of participants having work experience of between 4 and 6 years (83.7%, Table 1).

**Table 1:** Socio-demographic characteristics of farmers working in small-scale tomato farms, Cameroon, 2017 (*N* = 104)

Variables	Frequency *N* = 104	Percentage
Gender		
Male	90	86.5
Female	14	13.5
Marital status		
Single	19	18.3
Married	85	81.7
Age		
21–30	31	29.8
31–40	32	30.8
41–50	28	26.9
51–57	13	12.5
Level of education		
No formal	3	2.9
Primary level	25	24.0
Secondary level	65	62.5
Vocational training	9	8.7
Tertiary	2	1.9
Work experience		
1–3	17	16.3
4–6	87	83.7

### Participants’ farm sizes and description of products cultivated

The tomato farms were generally small, varying from 0.3 to 4.0 hectares (ha) with an average size of 1.1 ha. The majority (79.8%) of these farmers owned farms less than 1 ha as compared to 20.2% who owned more than 1 ha of the tomato farm. The main restraining factors for farmers to cultivate large surface areas were labour and capital. Although tomato fruit was the main product, most of these farmers also cultivated other crops such as peppers, green peppers, water melons, beans, green beans, green spices, carrots, maize, groundnuts, potatoes, cocoa, bananas, and cucumbers.

### Pesticide use in tomato farming

The analysis revealed that 18 pesticides were used on tomatoes by farmers in the western region of Cameroon with most (*n* = 15) of the pesticides enlisted on the homologated list of pesticides published by the Cameroon Ministry of Agriculture and Rural Development (MINADER). Three of these pesticides were not included in the homologated list. Out of the 15 pesticides enlisted on the Cameroon homologated list, 11 pesticides were obsolete, and four pesticides were not obsolete. The study analysis indicated that the most used fungicides in the study sites were maneb, mancozeb, metalaxyl, carbendazim, and thiophanate-methyl, while the most used insecticides were cypermethrin, imidacloprid, lambda cyhalothrin, chlorpyriphos-ethyl, endosulfan, and dimethoate. In addition, the frequently used herbicides were glyphosate, paraquat, and pendimethalin. All of the pesticides used are classified under the WHO chemical active ingredients hazards category class II (moderately hazardous) and class III (unlikely to cause hazards), as demonstrated in Table 2. The choice of pesticide used varied with season, area, and individual farmer.

**Table 2:** The major pesticides used by tomato farmers in the west region of Cameroon, 2017

Pesticides	Commercial name	Active ingredients (AI)	Chemical group	WHO chemical AI hazard classification
Fungicide	PLANTINEB 80 WP	Maneb 80%	Carbamate	II
	DITHANE M 45^a^, IVORY 80^a^	Mancozeb 640, 800 g/kg	Carbamate	II
	APRON 42 DS^a^	Metalaxyl 80 g/kg	Acylalanine	II
	CLEARY’S 3336^b^	Carbendazim	Benzimidazole	II
	TOPSIN M^b^	Thiophanate-methyl	Benzimidazole	II
Insecticides	AKITO 25 EC, CIGOGNE 12 EC^a^, CYPALM 50 EC^a^, CYPERCAL 50 EC, CYPLANDIM 260 EC^a^	Cypermethrin 12, 20, 50, 100 g/l	Pyrethroid	II
	PARASTAR 40EC	Imidaclopride 20 g/l + lambdacyhalothrine 20 g/l	Pyrethroid	II
	PYRIFORCE^a^	Chlorpyrifos-ethyl 600 g/l	Organophosphorus	II
	THIONEX 35R EC^a^	Endosulfan 350 g/l	Organochlorine	II
	DIMEX 400EC^a^	Dimethoate 400 g/l	Organophosphorus	II
Herbicide	Gramoxone^a^	Paraquat 200 g/l	Bipyridylium	II
	ROUND UP 360^a^	Glyphosate 360 g/l	Glycine derivative	III
	Corral G***†***	Pendimethalin 500 g/L	Dinitroaline	III

^a^ = Obsolete. † = ^b^Not enlisted on the Cameroon homologated list of pesticides. WHO classification class II = moderately hazardous and III = slightly hazardous

### The use and storage of pesticides by tomato farmers

**Fig. 1:**
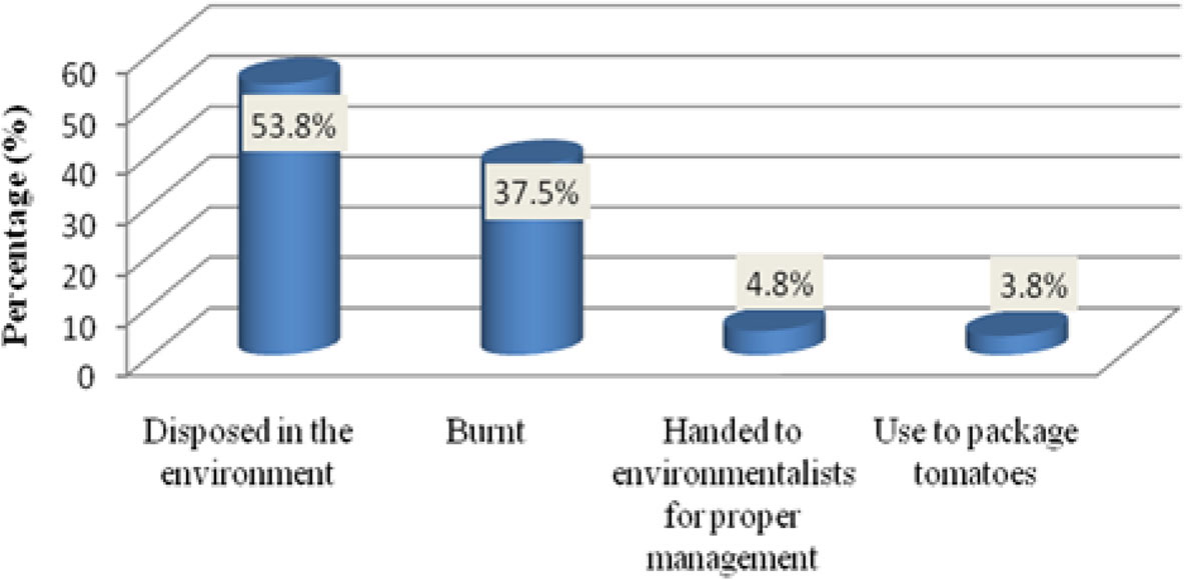
The management of empty pesticide sachets/containers

The duration between spraying pesticides and harvesting the tomato fruit for consumption ranged between 1 and 30 days, depending on the farmer and the type of pesticide used. The mean duration was 9.0 days. Regarding the storage of pesticides, 69.2% stored their pesticides on their farms, 26.9% stored the pesticides in their homes, and 26.9% stored the pesticides in their warehouse. Regarding the management of empty pesticide containers/sachets, 53.8% farmers discarded the waste on their farms, as compared to 37.6% who burned the containers themselves. Just 4.8% of farmers reported they handed their empty pesticide containers/sachets to environmentalists for proper management. Nevertheless, some (3.5%) farmers used the empty pesticide sachets as packaging for tomatoes, see Fig. 1.

### Training and safe practice of OHS among tomato farmers

Table 3 shows that 35.6% of the participants were trained on the use of PPE, while 64.4% have not received any form of OHS training. Thirteen participants (12.5%) reported they used safety boots during the spraying of pesticides as compared to 87.5% who did not. The use of safety glasses was also assessed, 31.7% used safety glasses when required while 68.3% did not. Regarding the use of gloves, the analysis revealed that 49.0% used gloves during work as compared to 51.0% who did not. Only 35.6% of the participants put on raincoats during the spraying of pesticides while 64.4% did not wear raincoats or any other form of protective clothing. The majority (99.0%) of the workers did not clean up their body immediately after using pesticides.

**Table 3:** Participants’ practices relating to the training and use of PPE, Cameroon, 2017 (*N* = 104)

Variables	Yes *N* (%)	No *N* (%)
Received training on the use of pesticides	37(35.6)	67(64.4)
Use of safety boots	13(12.5)	91(87.5)
Use of safety glasses	33(31.7)	71(68.3)
Use of gloves	51(49.0)	53(51.0)
Use of nose masks	69(66.3)	35(33.7)
Use of raincoats	37(35.6)	67(64.4)
Clean up the body immediately after the use of pesticides	1(1.0)	103(99.0)

### Participants’ work-related health complaints

Regarding participants’ work-related health problems/complaints, 24% complained of skin irritation after spraying of pesticides, 10.6% complained of backache, 9.6% reported nervous system injuries such as headache and dizziness, 16.3% reported visual problems, and 4.8% complained of respiratory difficulties (see Table 4).

**Table 4:** Work-related health complaints as reported by participants, Cameroon, 2017 (*N* = 104)

Health complaints	Yes *N* (%)	No *N* (%)
Skin irritation	25(24)	79(76.0)
Backache	11(10.6)	93(89.4)
Nervous system injury	10(9.6)	94(90.4)
Visual problems	17(16.3)	87(83.7)
Respiratory difficulty	5(4.8)	99(95.2)

### The association between the participant’s gender and the practice of OHS (*N* = 104)

Only the use of safety boots (*p* = 0.044) and the use of nose masks (*p* = 0.004) were significantly associated with the gender of the farmers (Table 5).

**Table 5:** The correlation between participants’ gender and the practice of OHS (*N* = 104)

Characteristics	Gender		Total *N* (%)	*X*^2^ (*p* value)
	Male *N* (%)	Female *N* (%)		
Use of safety boots				
Yes	81(89.0)	10(11.0)	91(100.0)	3.820(0.044)*
No	9(69.2)	4(30.8)	13(100.0)	
Use of safety glasses				
Yes	30(90.9)	3(9.1)	33(100.0)	0.792(0.373)
No	60(84.5)	11(15.5)	71(100.0)	
Use of gloves				
Yes	44(86.3)	7(13.7)	51(100.0)	0.006(0.938)
No	46(86.8)	7(13.2)	53(100.0)	
Use of nose mask				
Yes	55(79.7)	14(20.3)	69(100.0)	8.206(0.004)**
No	35(100)	0(0.0)	35(100.0)	
Use of raincoats				
Yes	33(89.2)	4(10.8)	37(100.0)	0.346(0.556)
No	57(85.1)	10(14.9)	67(100.0)	
Clean up the body immediately after the use of pesticides				
Yes	1(100.0)	0(0.0)	1(100.0)	0.157(0.692)
No	89(86.4)	14(13.6)	103(100.0)	

* = 0.01

** = 0.001

## Discussion

This study aimed at investigating the OHS conditions on the use of pesticides among farmers working in small-scale tomato farms in the western region of Cameroon. The majority of the farmers were males in their active age. This implies that the workforce in the small-scale tomato farming in the study sites is mostly male-dominated. This might be due to the hard and labourious work required, which might naturally limit the involvement of females and elderly people. The gender of the farmers also affected the use of safety boots and nose masks (*p* < 0.05).

These results confirmed the findings of Tarla et al. [14] who reported that the majority of small-scale tomato farmers in the western region of Cameroon were males. Women assisted their husbands in activities that did not require a lot of energy, such as transplanting and harvesting of tomato fruits. Similarly, another study conducted by Tandi et al. [15] in evaluating the perception of small-scale tomato cultivators on pesticide usage and practices in Buea, in the southwest region of Cameroon revealed that 96.7% of tomato farmers were males.

Regarding the level of education, the study revealed that secondary school was the highest level of education attained by the majority of tomato farmers in the study area. It has been argued that being educated increases access to information, training, and communication materials, enables a better awareness of various workplace hazards, and ensures an understanding of safe work procedures and a better propensity to develop a positive attitude towards OHS at work. The findings confirm results of a study by Tandi et al. [15] that made known that most tomato farmers in Cameroon had no formal education. This study corroborates the results of Kenko et al. [16] that revealed that the majority of local farmers in the southwest region of Cameroon attained only secondary education. The findings are, however, not in line with the study conducted by Gesesew et al. [17] in southwest Ethiopia which revealed that about two thirds (67.5%) of participants could read and understand labels/instructions from the pesticide container if written in the local language.

The analysis revealed that fungicides, insecticides, and herbicides were the pesticides used on tomatoes in the area of study. In addition, insecticides were the most used while herbicides were the least used pesticides. This implies that fungi, insects, and herbs are the major hindrance to production of tomatoes in the study area. This study findings corroborates a study by Tandi et al. [15] conducted in the southwestern region of Cameroon that found that insecticides, fungicides, and herbicides are the most frequently used pesticides by tomato cultivators to control pests, with insecticides being the most used. Also, the least used pesticides according to Tandi et al. [15] was herbicide, this was probably due to the fact that most farmers did manual weeding of their farms with their family members or friends using hands, cutlasses, and hoes on the smaller farms.

The majority of tomato farmers burned empty pesticide containers or disposed it in the fields. The indiscriminate disposal of these containers in the field could cause the accumulation of pesticide in soil and water sources, as was previously detected in a sample of irrigation water [18]. Some pesticides’ active ingredients might not decompose in the soils or water and can therefore be attributed to as the cause for pesticide residues in tomatoes. This practice of indiscriminate disposal of pesticide containers in the fields have been reported in Tanzania [19], as well as Khan, Shabbir, Majid, Naqvi, and Khan [20] in Arumeru-Tanzania and Pakistan.

The current findings showed that the majority of the tomato farmers in the study area have poor OHS practices as a result of inadequate OHS training and use of PPE. Good practices in OHS generally require respondents to comply with OHS practices during the execution of their duties and lead to more positive health and safety culture among the workers and can significantly reduce both injury rates and costs at the workplace [21]. Comparable preceding studies conducted in Cameroon and the Philippines revealed that the use of PPE was rare among participants [22, 23]. Asongwe et al. [22] revealed that 95% of farmers in Bamenda Municipality of Cameroon do not protect themselves during pesticide applications. In addition, Palis et al. [23] made known that those Filipino farmers believe in immunity, meaning that the youths were not susceptible to the adverse health effects of pesticides. Consequently, PPE were not important to them [23]. The present study findings contradict the result of a study by Negatu et al. [24] conducted in Ethiopia, which reported that 100% of participants used PPE.

Regarding the work-related problems sustained by farmers, current findings showed that the common work-related problems were skin irritation, backache, nervous system injury such as headache and dizziness, visual problems, and respiratory difficulties after spraying pesticides. Generally, farmers believe that pesticide poisoning symptoms are ordinary so they get used to them [23]. Comparable studies carried out in Tanzania [25] and the Ivory coast [26] reported that pesticide applicators were likely to accept a certain level of illness as an expected and normal part of farming and never reported the symptoms to health centres for prescribed medical assistance.

### Limitation of the study

The current findings are limited by the cross-sectional nature of the study design, with tomato farmers recalling information and increasing the possibility of misclassification of exposure and health presentation. Nevertheless, by retraining the researcher’s analysis to farmers cultivating tomatoes limited the potential for exposure misclassification. Furthermore, there is the limitation of analysis of blood-pesticide residue of the people that were exposed to pesticides due to the unavailability of facilities.

## Conclusion and recommendations

Findings confirmed that working in small-scale tomato farming might be unsafe, due to poor OHS conditions leading to the predisposing of farmers to the risk of work-related health problems. Exposure to occupational hazards can be significantly reduced if the required PPE are used. Increasing farmers’ awareness on good practices for pesticide application and the strengthening of food safety control services for pesticide control as measures to prevent and protect public health against pesticides is recommended. This study concentrated on pesticides used within the western region of Cameroon; future studies should examine blood-pesticide levels, given the high exposure in other regions of the country, to enable the development of a national strategic plan to address pest control and pesticide use.

## Data Availability

All data generated or analysed during this study are included in this published article [and its supplementary information files].

## Abbreviations

CNS: Central nervous system
FAO: Food and Agriculture Organization
GDP: Gross domestic product
MINADER: Ministry of Agriculture and Rural Development
OHS: Occupational health and safety
PPE: Personal protective equipment
WHO: World Health Organization

## References

[1] Johnston BF, Mellor JW (2007). The role of agriculture in economic development. AgEcon Rev.

[2] Jean Sonchieu, Benoit Ngassoum Martin, Edouard Nantia Akono, Srivastava Laxman Prasad (2017). Pesticide Applications on Some Vegetables Cultivated and Health Implications in Santa, North West-Cameroon. International Journal of Agriculture & Environmental Science.

[3] World Health Organization (WHO) (1986). Informal consultation on planning strategy for the prevention of pesticide poisoning.

[4] Pretty J, Bharucha ZP (2015). Integrated pest management for sustainable intensification of agriculture in Asia and Africa. Insects..

[5] Yanez L, Ortiz D, Calderon J, Batres L, Carrizales L, Mejia J, Martinez L, Garcia-Nieto E, Diaz-Barriga F (2002). Overview of human health and chemical mixtures: problems facing developing countries. Environ Health Perspect.

[6] Carvalho FP (2006). Agriculture, pesticides, food security and food safety. Environ Sci Pol.

[7] Mancini F, Van Bruggen AH, Jiggins JL, Ambatipudi AC, Murphy H (2005). Acute pesticide poisoning among female and male cotton growers in India. Int J Occup Environ Health.

[8] Oesterlund AH, Thomsen JF, Sekimpi DK, Maziina J, Racheal A, Jors E (2014). Pesticide knowledge, practice and attitude and how it affects the health of small-scale farmers in Uganda: a cross-sectional study. Afr Health Sci.

[9] FAO. Prevention and disposal of obsolete and unwanted pesticide stocks in Africa and the Near East - Fourthconsultation meeting. Rome: Food and Agriculture Organization of the United Nations; 1999.

[10] Asante BO, Osei MK, Dankyi AA, Berchie JN, Mochiah MB, Lamptey JNL, Haleegoah J, Bolfrey-Arku Osei KG (2013). Producer characteristics and determinants of technical efficiency of tomato based production systems in Ghana. J Dev Agric Econ.

[11] Litchfield MH (2005). Estimates of acute pesticide poisoning in agricultural workers in less developed countries. Toxicol Rev.

[12] Jeyaratnam J (1990). Acute pesticide poisoning: a major global health problem. World Health Stat Q.

[13] London L, Bailie R (2001). Challenges for improving surveillance for pesticide poisoning: policy implications for developing countries. Int J Epidemiol.

[14] Tarla DN, Manu IN, Tamedjouong ZT, Kamga A, Fontem DA (2015). Plight of pesticide applicators in Cameroon: case of tomato (*Lycopersiconesculentum* Mill.) farmers in Foumbot. JAES..

[15] Tandi TE, Wook CJ, Shendeh TT, Eko EA, Afoh CO (2014). Small-scale tomato cultivators’ perception on pesticides usage and practices in Buea Cameroon. Health..

[16] Kenko NDB, Asanga BFP, Ngameni TN, Mpoame M (2017). Environmental and human health assessment in relation to pesticide use by local farmers and the Cameroon development corporation (CDC), Fako division, South-West Cameroon. ESJ..

[17] Gesesew HA, Woldemichael K, Massa D, Mwanri L (2016). Farmers knowledge, attitudes, practices and health problems associated with pesticide use in rural irrigation villages, Southwest Ethiopia. PLoS One.

[18] Damalas Christos, Koutroubas Spyridon (2016). Farmers’ Exposure to Pesticides: Toxicity Types and Ways of Prevention. Toxics.

[19] Lekei EE, Ngowi AV, London L (2014). Farmers’ knowledge, practices and injuries associated with pesticide exposure in rural farming villages in Tanzania. BMC Public Health.

[20] Khan DA, Shabbir S, Majid M, Naqvi TA, Khan FA (2010). Risk assessment of pesticide exposure on health of Pakistani tobacco farmers. J Expo Sci Environ Epidemiol.

[21] Wolska L, Namies’nik J (2007). Quality and environmental management systems in Polish shipbuilding industry. Methods of implementation. Pol J Environ Stud.

[22] Asongwe GA, Yerima BPK, Tening AS (2014). Vegetable production and the livelihood of farmers in Bamenda municipality. Cameroon Int J Curr Microbiol App Sci.

[23] Palis GF, Warburton RJH, Mahabub H (2006). Our farmers at risk: behaviour and belief system in pesticide safety. J Public Health.

[24] Negatu B, Kromhout H, Mekonnen Y, Vermeulen R (2016). Use of chemical pesticides in Ethiopia: a cross sectional comparative study on knowledge, attitude and practice of farmers and farm workers in three farming systems. Ann Occup Hyg.

[25] Ngowi AVF, Mbise T, Ijani ASM, London L, Ajayi OC (2007). Pesticides use by smallholder farmers in vegetable production in northern Tanzania. Crop Prot.

[26] Ajayi OC. Pesticide use practices, productivity and farmer’s health: the case of cotton-rice systems in Cote d’Ivoire, West Africa. Hannover, Germany: A publication of the Pesticide Policy Project; 2000. p. 172. (Special Issue Publication Series, No. 3).

